# ACTN3 Genotypes and Their Relationship with Muscle Mass and Function of Kosovan Adults

**DOI:** 10.3390/ijerph18179135

**Published:** 2021-08-30

**Authors:** Arben Boshnjaku, Ermira Krasniqi, Harald Tschan, Barbara Wessner

**Affiliations:** 1 Centre for Sport Science and University Sports, University of Vienna, Auf der Schmelz 6, 1150 Vienna, Austria; harald.tschan@univie.ac.at (H.T.); barbara.wessner@univie.ac.at (B.W.); 2 Faculty of Medicine, University “Fehmi Agani” in Gjakova, Ismail Qemali n.n., 50000 Gjakovë, Kosovo; 3 Department of Nutritional Sciences, Faculty of Life Sciences, University of Vienna, Althanstraße 14, 1090 Vienna, Austria; ph.ermirakrasniqi@gmail.com; 4 Research Platform Active Ageing, University of Vienna, Althanstraße 14, 1090 Vienna, Austria

**Keywords:** ageing, muscle strength, physical performance, genotypes, ACTN3 R577X

## Abstract

Maintaining muscle mass and function is important throughout the lifestyle. While environmental factors such as physical activity and healthy nutrition are well investigated, the contribution of genetic factors is still controversial. Therefore, we aimed to investigate the impact of a common ACTN3 polymorphism (rs1815739) on body composition, handgrip strength, knee extensor peak torque, and physical performance (gait speed, 30-s arm curl, 30-s chair stand) in Kosovan adults. In total, 308 participants (160 females and 148 males, age range from 40 to 91 years) took part in this cross-sectional study. Genomic DNA was extracted from saliva and assessed for ACTN3 genotype distribution (41.5% of RR, 53.9% of RX and 4.6% of XX). Genotype allocation did not account for differences in any of the variables. Interestingly, female XX carriers were taller (*p* = 0.025) and had a higher isokinetic knee extension peak torque (*p* = 0.024) than the RX+RR group. In males, XX carriers were also taller (*p* = 0.049) and had a lower BMI (*p* = 0.026), but did not differ in any of the strength and performance parameters. These results indicate that the ACTN3 R577X polymorphism might exert a sex-specific impact on knee extensor peak torque and BMI.

## 1. Introduction

Muscle mass and function have been shown to decline with increasing age, especially beyond 50 years, where a yearly decline of about 1–2% in muscle mass, 1.5–5% in muscle strength, and 3.5% in muscle power can be expected [1,2]. The importance of strength and power are emphasized particularly in maintaining the quality of life and independence for daily activities of life, as well as prohibiting falls, fractures, morbidity, and even mortality. Therefore, it is not surprising that these parameters are also used as diagnostic factors for sarcopenia, a progressive and generalized skeletal muscle disorder associated with ageing and immobility [3,4].

Many changes underlying the age-associated decay of the musculoskeletal system can be understood at the cellular and molecular level [5]. Muscle fiber numbers and size decrease with age with a disproportional shrinking of fast-twitch type II fibers being especially important for power-related muscle traits [6]. In addition, genetic factors have been studied for their potential relation with power-related muscle traits. However, relatively few of the investigated genotypes have been proven to significantly contribute towards a variation in muscle phenotypes [7]. The identification of genetic variants with the potential to influence muscle phenotypes is of interest not only for elite athletes, but also for its potential to identify persons at risk and to develop individualized therapeutic strategies [8,9]. With respect to muscle function, much attention has been paid to the ACTN3 gene, expressing one of two major components of the contractile apparatus at the Z-line (alpha-actinin-3 protein) and being found only in fast twitch type II fibers [10]. A common null polymorphism of ACTN3 (R577X-rs1815739) is responsible for the fact that individuals homozygous for the X allele are unable to express α-actinin-3 in contrast to those with the RX or RR genotype [11]. Despite the growing body of evidence suggesting the potential modifying effect of ACTN3 genotypes [12] on muscle strength [13,14,15,16,17], mass [13,18,19,20], power [13,21], and functional performance [17,22,23,24] in older populations, inconsistency exists amongst studies [7,19,25,26,27,28]. Thereby, several studies go in line with the physiological expectations and show an increased muscle mass [18,20], strength [14,16,23], power [22], or physical performance [22] in individuals carrying the R allele. However, these results are contrasted by studies revealing better results for XX individuals [17,21], studies with mixed results for different parameters [13] or with respect to trainability [16,19].

Furthermore, the frequency distribution of the ACTN3 XX genotype differs across different populations, while averaging from approximately 25% in Sweden, 21.6% in Italy, 18.3% in Greece, 10.4% in Lithuania, to as low as 1% in Kenya, 1% in Bantu, and 0% in Nigeria (Yoruba) [29]. Therefore, the main aim of this study was to acquire population-specific ACTN3 genotype distributions in Kosovo and to investigate their distinct characteristics and sex-based relationships to various parameters of muscle mass, strength, and function.

## 2. Materials and Methods

### 2.1. Participants

Males and females with Kosovan nationality aged 40 years and above and living in or in the surroundings of Prishtina, the largest of seven regions of Kosovo with a total of 477,312 inhabitants, were eligible to take part in the study [30]. Recruitment involved national broadcasting as well as written and verbal announcements in the Kosovo Pensioners’ Association and Prishtina Nursing Homes in Prishtina and Fushë Kosova (neighboring municipalities). Participants were allowed to bring their partners. Exclusion criteria represented health conditions that would not allow to perform exercise as screened by physical activity readiness questionnaire (PAR-Q) [31], the presence of chronic diseases that could impair the measurements of physical performance such as serious cardiovascular diseases (i.e., decompensated chronic heart failure, severe or symptomatic aortic stenosis treated, pacemakers, unstable angina, cardiac arrhythmias, diabetic retinopathy) or any other similar conditions that could prohibit subjects performing measurements [32]. Inclusion and exclusion criteria were assessed by two health professionals at the entrance examination.

Following detailed explanations of the testing procedures, written informed consents were obtained from all participants. The study protocol was reviewed and approved by the Ethical-Professional Committee of the University Clinical Centre of Kosovo (no. 1246/15.09.2016), whereas all procedures were performed in accordance with the guidelines of the Declaration of Helsinki for Human Research.

Data were collected during two separate visits by trained assessors. At first health status, socioeconomic, nutritional, and physical activity levels together with anthropometric measurements, muscle strength, isokinetic peak torque and gait speed, were assessed. On the second visit, saliva samples for isolating DNA were collected from the study participants.

### 2.2. Anthropometric, Muscle Strength, Isokinetic Peak Torque, and Physical Performance

#### 2.2.1. Data Collection and Anthropometric Assessments

Following the previously set protocol, data collection was organized at the Sports Medicine Laboratory in Universi College in Prishtina. Test–retest reliability of all the used assessments was evaluated and described previously [33]. Anthropometric measurements were performed in accordance to the International Standards for Anthropometric Assessment [34] by the same research team during the entire data collection period.

Height was measured barefoot wearing light indoor clothing, using a portable stadiometer and following the stretch stature method (DT05L, Kinlee, Zhongshan Jinli Electronic Weighing Equipment Co. Ltd., Guangdong, China). Weight and body composition (including whole-body skeletal muscle mass (SMM) and fat mass) were measured using a segmental multifrequency bioelectrical impedance analyser (BIA) (Inbody 770 device, Biospace Co., Ltd., Seoul, Korea) with data output calculated by manufacturer’s algorithm. This process required the participants to hold the hand electrodes while standing upright above the tactile foot electrodes. Furthermore, body mass index (BMI) was determined as body mass by height squared (expressed as kg/m^2^) [35].

#### 2.2.2. Handgrip Strength and Isokinetic Knee Extensor Peak Torque Assessments

Isometric handgrip strength was assessed in a sitting position through recording the best of two trials by maximally squeezing the adaptable dynamometer (JAMAR, Patterson Medical, Warrenville, IL, USA) with the self-reported dominant hand (1 min rest in between trials) [36]. Isokinetic dynamometry [37] was used to assess the absolute and relative knee extensors isokinetic concentric peak torque at 60°/s unilaterally on the self-reported dominant side (Biodex System 4 Pro, Biodex Medical Systems, Inc., Shirley, NY, USA). During the assessment, participants were fixed on the device’s chair with straps over the shin, thigh, pelvic, and crossed above upper torso, while holding the supporting sideway handles [38].

#### 2.2.3. Functional Performance Measurements

Physical performance was assessed through six-meter gait speed (m/s) for usual walking speed, 30-s chair stand test (reps) for lower body strength (endurance) and 30-s arm curl test (reps) for upper body strength (endurance) [36,39]. Gait speed was assessed over six meters on a course 10 m in length, using the first two meters for acceleration and the last two meters for deceleration. Time to pass the middle six meters was taken by means of a stopwatch. The usage of a cane was only allowed if a participant expressed the absolute necessity to use it while performing (n = 4). The 30-s chair-stand test was administered as described by Jones and Rikli [39] by instructing the participants to stand up and sit down as often as possible within 30 s from a 46 cm high armless chair placed against the wall. Similarly, the 30-s arm curl test was used to assess the number of repetitions for weight-loaded (5 pounds for woman and 8 pounds for men) elbow flexion and extension of the self-reported dominant arm, while the participants were sitting on an armless chair [39]. In both tests (30-s chair-stand and arm curl), the tester stood next to the participant while signaling the starting and ending time points and counting the number of successful repetitions, including the very last attempt if more than 50% of the range of motion was mastered before the timeline.

### 2.3. Genetic Analyses

Participants were asked to refrain from eating, drinking, smoking, brushing teeth, or chewing gum for 30 min before the sample collection. Every participant spit about 2 mL of saliva in a tube prefilled with 2 mL stabilization buffer. The tubes were thoroughly mixed and stored at −20 °C for not longer than three months. After finalizing sample collection, they were sent for further analyses to the Laboratory of Molecular Exercise Physiology at the Centre for Sport Sciences and University Sports, University of Vienna, Austria. Genomic DNA was extracted from the saliva samples using GeneFiX™ Saliva-Prep DNA Isolation Kit (Isohelix, Kent, UK) following the manufacturer’s instructions. Quantity (58.07 ± 57.02 ng/mL) and quality (A_260_/A_280_: 1.77 ± 0.17) of DNA were checked on a NanoDrop spectrophotometer (Peqlab, Erlangen, Germany). From the isolated DNA samples, quantity, and quality of 282 samples were high enough to perform genotyping. Genotyping was performed using real time PCR (QuantStudio 7 Flex real-time PCR System, ThermoFisher Scientific, Vienna, Austria) and a commercially available TaqMan^TM^ SNP genotyping assay to detect the ACTN3 R577X polymorphism (rs1815739, C_590093_1, ThermoFisher Scientific, Vienna, Austria). According to the manufacturer’s instruction, a reaction mix was prepared containing 2.50 µL of 2X TaqMan^TM^ Master Mix and 0.25 µL of 20X Assay Working Stock per well. Each DNA sample was diluted with DNase free water to deliver 20 ng of DNA per well. Total reaction volume was set to 5 µL and reactions were performed on a MicroAmp™ Optical 384-Well Reaction Plate (ThermoFisher Scientific, Vienna, Austria). Samples were analyzed in triplicates and a no-template control with DNase-free water instead of a DNA sample was run on each plate. A pre-PCR plate read was performed to record the background fluorescence of each well. Cycling conditions involved 95 °C for 10 min for polymerase activation, and 40 cycles with alternating steps at 95 °C for 15 s followed by 60 °C for 1 min. Finally, fluorescence was detected at the post-PCR plate read and used to determine the genotypes of the samples.

### 2.4. Statistical Analyses

All statistical evaluations were performed by the SPSS statistical package version 26.0. As normal distribution was violated for many of the parameters, data were expressed as medians and interquartile ranges (75th minus 25th percentile) in case of continuous variables and as absolute numbers or percentages for frequencies of categorical variables. The non-parametric Mann–Whitney U test was used to determine potential differences between two groups, whereas Kruskal–Wallis test was used to evaluate differences between the three genotypes. *p* values below 0.05 were considered as being statistically significant.

Hardy–Weinberg equilibrium (HWE) was calculated using chi-squared (χ^2^) statistics with one degree of freedom, comparing the observed distribution of genotypes with the distribution of genotypes expected from applying the Hardy–Weinberg equilibrium assumption (using the online application: https://ihg.gsf.de/cgi-bin/hw/hwa1.pl (accessed on 30 August 2019)).

## 3. Results

In total, 308 participants (160 females and 148 males, age range from 40 to 91 years) could be recruited via media (n = 68) or the Kosovo Pensioners’ Association and Prishtina Nursing Homes (n = 240) between September 2016 and November 2017. From these participants, 299 persons of Caucasian descent agreed to provide a salivary DNA sample. Unfortunately, further 17 samples (5.7%) could not be genotyped based on the low quality of the DNA sample. Therefore, 282 participants (91.6%) were included in the final analyses. Participants’ general characteristics for the total genotyped sample and separated by sex are summarized in Table 1.

Differences between males and females in the genotyped participants were observed for age (U = 13667.5, Z = 5.499, *p* < 0.001), body weight (U = 11783.0, Z = 2.742, *p* = 0.006), height (U = 17689.5, Z = 11.402, *p* < 0.001), BMI (U = 6543.5, Z = −4.922, *p* < 0.001), SMM (U = 17413.0, Z = 11.149, *p* < 0.001), body fat mass (U = 4988.0, Z = −7.128, *p* < 0.001), body fat percentage (U = 2563.0, Z = −10.695, *p* < 0.001), handgrip strength (U = 16963.0, Z = 10.319, *p* < 0.001), relative handgrip strength (U = 15954.5, Z = 8.844, *p* < 0.001), knee extensors peak torque (U = 14559.5, Z = 6.803, *p* < 0.001), relative knee extensors peak torque (U = 14007.5, Z = 5.996, *p* < 0.001), and gait speed (U = 12210.0, Z = 3.367, *p* = 0.001), whereby higher values were obtained for males except for BMI and fat mass. No differences were found for 30-s arm curl (U = 10454.0, Z = 0.801, *p* = 0.423) and 30-s chair stand test (U = 11079.0, Z = 1.721, *p* = 0.085).

ACTN3 genotype distribution of the total sample was 41.5% RR (n = 117), 53.9% RX (n = 152), 4.6% XX (n = 13) whereas the allelic frequencies were 0.68 and 0.32 for the R and X alleles, respectively. Noteworthy, the genotype distribution for the entire cohort did not meet HWE (χ^2^ = 17.09, *p* < 0.001), whereby the inbreeding factor (F) was detected to be negative (F = −0.248). Participants’ characteristics for the ACTN3 genotypes (RR, RX, and XX), as well as the dominant (RR + RX) and recessive (RX + XX) models with the matched genotypes (XX and RR, respectively) are shown in Table 2 for total participants, Table 3 for females and Table 4 for males. The three genotype groups did not differ in any of the measured variables, neither in the total cohort nor separated by sex (*p* > 0.05) as evidenced by Kruskal–Wallis tests.

For the dominant model, no differences were detected between RR+RX and XX for any of the measured parameters (*p* > 0.05) besides age with participants carrying the XX genotype being slightly older (U = 2323.5, Z = 2.002, *p* = 0.045). This difference was not confirmed when the total group was separated by sex (females: U = 763.0, Z = 1.676, *p* = 0.094; males: U = 444.5, Z = 1.473, *p* = 0.141). However, within the female subgroup, the XX carriers (n = 8, 5.4%) were also taller (U = 830.5, Z = 2.248, *p* = 0.025) and had a higher isokinetic knee extension peak torque (U = 832.5, Z = 2.261, *p* = 0.024). Furthermore, the median skeletal muscle mass was higher, though not significantly (U = 787.0, Z = 1.878, *p* = 0.060). In males, XX carriers (n = 5; 3.8%) were again taller (U = 486.5, Z = 1.972, *p* = 0.049) and had a lower BMI (U = 132.0, Z = −2.224, *p* = 0.026). However, these data need to be regarded with care given the very low number of XX carriers in the total population.

For the recessive model, RR carriers (N = 117, 41.5%) did not differ from RX+XX carriers in any of the variables, neither in the total cohort nor separated by sex (*p* > 0.05).

## 4. Discussion

This study aimed to evaluate the influence of ACTN3 genotypes (RR, RX, and XX) on muscle strength, mass, isokinetic knee extensor peak torque and functional performance in a population of mature adults from Kosovo, a developing lower income country. No significant differences could be detected between the three genotypes neither in the total population nor specifically for men and women. Notwithstanding, differences were observed in females when comparing the XX to the RR+RX group. In this dominant model, XX carriers were taller with a higher isokinetic knee extensors peak torque (60°/s) and a tendency to higher relative isokinetic knee extensor peak torque and muscle mass.

These findings support the hypothesis that the ACTN3 R577X polymorphism would influence force-generating capacity of knee extensors in a sex-specific manner. Similar to our findings, Delmonico and colleagues [21] have shown that older females with the XX genotype were in favor to have a higher absolute and relative knee extensor concentric peak power in comparison to the RR and RX groups while no differences in absolute or relative peak power were observed between ACTN3 genotype groups in men [21]. Based on the physiological role of ACTN3 in force transmission these results are unexpected. Irrespective of baseline values the authors showed an advantage for RR homozygotes in peak power response to a 10 weeks lasting strength training [21]. While female-specific associations between ACTN3 genotype and knee extension peak torque were confirmed in another study, the concrete outcome differed as α-actinin-3-deficient women displayed lower knee extensor shortening and lengthening peak torques compared to women with RX+RR genotypes. Furthermore, the women homozygous for XX had a lower level of total fat free mass and lower limb fat free mass [13]. However, differently to the BIA technique (by Inbody device) used in our study, Walsh et al. used the dual energy X-ray absorptiometry (DEXA) to measure the whole-body soft tissue composition. Nevertheless, both techniques have shown high correlation degree in muscle mass assessment [40] and are both recommended to be used in research [3]. Potential differences in sample size and age may eventually explain the divergent results within these studies. While age and sample size were similar between our study (308 participants, 160 females and 148 males, 40–91 y) and the one by Delmonico and colleagues (157 Caucasian participants, 71 males and 86 females, 50–85 y) [21], the study by Walsh and colleagues [13] included a higher number and also younger participants (848 volunteers, 454 males and 394 females, 22–90 y). Nevertheless, Walsh et al. [13] observed the same outcomes even after calculation in a subcohort of females aged above 50 years. Up to now no mechanistic explanation for these unexpected and dichotomous findings can be given although it might be important to discriminate between studies in highly trained athletes, where elite sprint athletes have significantly higher frequencies of the 577R allele than do controls [41] and those with rather untrained (and older) populations. In addition, it has been suggested that subjects possessing the ACTN3-deficient genotype (XX) had lower baseline creatine kinase levels, a marker of muscle damage, compared with the heterozygotes [42]. This might affect the response to strength training. If it also would affect general strength or power measures is still under investigation and to our knowledge no data exist in older adults.

Most studies showing ACTN3 R577X polymorphism’s association with muscular strength and power primarily concentrate on physical performance and trainability rather than ageing and physical fitness of older people. Yet, the ACTN3 polymorphism has also been described as an evolutionary tool for improving the energy economy during locomotion [43]. A recent meta-analysis, estimating the association of ACTN3 R577X polymorphism with elite power sports, emphasized the importance of understanding the potential genetic impact together with the modifying effects of ACTN3 R577X genotype on organisms’ phenotypes and consequently on conditions such as muscle deterioration (sarcopenia) [20]. Some studies [14,44] have shown an association between the ACTN3 genotype and sarcopenia-related declines in muscle phenotypes in older adults. Outcomes from a recent study suggest that active women aged 75 and above carrying the R allele might have a higher risk for sarcopenia in comparison to the XX carriers [17], which somehow fits to the tendencies observed in our study. However, it is very likely that various genotypes would interact in modulating muscle-related phenotypes. A study investigated the impact of ACE I/D and ACTN3 R577X polymorphisms on maximal strength of the arm and leg muscles, muscle power performance (counter-movement jump) and functional capacity (sit-to-stand test) subsequent to 12 weeks of high-speed power training. Significant differences in all parameters in response to high-speed power training were detected between the power (ACTN3 RR + RX & ACE DD) and the ‘non-power’ muscularity-oriented genotypes (ACTN3 XX & ACE II + ID)] [45]. In this respect, the underlying cause of the obtained results might be a result of gene × gene or gene × environment interactions which need to be elucidated in further studies.

The rather low prevalence of ACTN3 XX genotype found in this Kosovan study population (4.6%) present a novelty within the world-wide human populations’ prevalence based on the countries mean geographical latitude [29]. In fact, countries positioned in similar coordinates (Kosovo latitude 42.60°) have shown rather different prevalences, varying between 24% (Japan, 41.50°), 21.6% (Italy, 42.61°), 19.9% (Spain, 40.64°), and 18.3% (Greece, 40.00°) [29]. Nevertheless, our findings are consistent with a prevalence study from six neighboring Balkan countries. Even though a relatively small number of participants (total n = 483) have been genotyped, a generally lower representation of the ACTN3 XX genotype, varying from 6.0% in North Macedonia, 6.7% in Montenegro, 12.5% in Albania, 13.1% in Croatia, 13.6% in Serbia, and 17.7% in Bosnia and Herzegovina has been confirmed [46]. From these, North Macedonia, Montenegro, Albania, and Serbia are land bordered with Kosovo. This underrepresentation below the reference latitude populations opens a gap for further discussion within the field. This is important especially since the allelic frequencies from our study (0.68 and 0.32 for the R and X alleles, respectively) are comparable to other European (0.58 and 0.42, respectively) [47] and Balkan populations (from 0.59 and 0.41 in Serbia, 0.66 and 0.34 in Albania, to 0.79 and 0.21 in Montenegro) [46]. A factor that seems to be very specific for Kosovo and could be an important factor for genetic studies, is the fact that compared to neighboring countries—such as Albania, Macedonia, and Serbia—Kosovo has had among the lowest number of immigrants in South East Europe. It remained outside the so-called Balkan Route [48]. According to the 2011 Population Census data, in Kosovo there were 128,808 immigrants, which comprised 7.4% of the population. Around 97% of immigrants had Kosovan citizenship; therefore, the vast majority of immigrants in fact were returnees to their country. Taking into account the citizenship of its residents, Kosovo seems to be a fairly homogeneous country [49].

Another aspect that undoubtedly presents an issue of great scientific interest as well as a serious public health concern is the very high level of overweight (42.9%) and obesity (39.4%) found for the present study population. This necessarily needs to be thoroughly analyzed and further explored in subsequent studies taking potential co-variates (e.g., socio-economic status, educational level, health status) into consideration. However, the higher levels of overweight and obesity could be also connected to the low level of participants homogenous for the XX genotype as this variant may affect body metabolic phenotypes [50]. Amongst the few studies exploring this phenomenon, Houweling et al. [50] were suggesting that the XX genotype may be protective against obesity in knockout mouse model. Lower body weight and fat-free mass was also associated with the XX genotype in female elite ballerinas aged 18–39 years, but not in the control group which had a higher prevalence of the XX genotype (20.2% in contrast to 24.7%) [51]. Furthermore, it has been shown that women homozygous for XX displayed lower levels of weight, BMI, body fat free mass (FFM), lower limb FFM and fat mass, compared to other genotypes [13]. Therefore, findings from our study such as the lower BMI of the male XX carriers, together with the simultaneous low prevalence of XX homozygotes and high number of overweight and obese participants support an association between the ACTN3 genotype and obesity.

Another important issue to be mentioned is the fact that this study was not in HWE. This violation might be affected by several factors, starting from genotyping, inbreeding, small group of participants, mutation, etc. [52]. The assay quality with the successful genotyped samples was satisfyingly high (94%), thereby excluding a methodological issue with the genotyping. For recruitment, a multidirectional approach has been used as described in the methods section. The negative inbreeding factor (F = −0.248) suggests an excess of heterozygotes [53]. Participants were allowed to invite their partners in the study, but the degree of heredity (family relations) between different participants was not assessed. Given the relatively low number of inhabitants in the administrative region of Prishtina (477,312) within a small country (1,739,825 inhabitants), and with a low percentage of people aged 60 [30] and above closer relationships could have contributed to the observed outcomes.

Although the study has been performed with utmost care, its limitations need to be mentioned. First, the low baseline allele frequency of 577X within our sampled population (4.6%) could reduce the effective power of the study, to potentially obscuring the genetic association with muscle mass and function. In fact, this encountered outcome (lower than the worldwide average of 18% [12]) was both interesting and intriguing. Another issue would be the very high percentage of overweight and obesity in general population, which could as well mask the outcome measures. Furthermore, the violation of HWE is another limitation that needs to be mentioned. Finally, future epidemiological studies are definitely needed, which could aim to balance the number of participants with respect to the different genotypes, in order to overcome the statistical limitations inherent with low sample sizes. Additional data with respect to sarcopenia prevalence or consequent risk of falls and associated illnesses would be very important to interpret the genetic data in the specific environmental context.

## 5. Conclusions

This study shows that from a convenient sample of Kosovo mature adults, there were no direct significant associations between the three ACTN3 genotypes (RR, RX, and XX) with muscle strength, mass, isokinetic peak torque and functional performance in neither total population, nor sex-specifically). Distinctive observations hint to sex-specific outcomes as female carriers of the XX genotype showed significantly higher isokinetic knee extensors peak torque (60°/s) and male carriers of the XX genotype had a significantly lower BMI than the RR + RX genotype groups. However, this study also highlights the need for further studies as understanding the multifactorial relationship between genotypes, various covariates and the development of sarcopenia is the key for developing accurate diagnostic tools for an earlier detection of sarcopenia and similar related conditions.

## Figures and Tables

**Table 1:** Participants’ characteristics.

Variable (Units)	Genotyped Sample N = 282	Females N = 149	Males N = 133	*p*-Value
Age (years)	67.6 (60.7–72.7)	65.2 (57.6–70.2)	70.3 (66.4–74.3)	<0.001
Body weight (kg)	77.6 (70.8–86.5)	76.8 (68.4–83.9)	79.8 (73.4–88.1)	0.006
Height (m)	1.64 (1.58–1.72)	1.59 (1.54–1.63)	1.72 (1.66–1.75)	<0.001
BMI (kg/m^2^)	28.98 (26.40–32.21)	30.16 (27.44–34.05)	27.53 (25.06–30.03)	<0.001
Skeletal muscle mass (kg)	25.8 (22.6–30.4)	23.3 (21.0–25.3)	30.7 (27.1–34.6)	<0.001
Fat mass (kg)	28.4 (21.6–35.7)	32.5 (26.7–39.4)	24.4 (18.8–29.1)	<0.001
Fat percentage (%)	36.9 (29.2–44.2)	43.3 (37.9–48.2)	30.1 (24.7–35.1)	<0.001
Handgrip strength (kg)	29.3 (24.1–37.5)	25.7 (21.7–28.6)	37.4 (30.9–42.8)	<0.001
Relative handgrip strength (kg/kg)	0.39 (0.31–0.48)	0.33 (0.28–0.39)	0.45 (0.39–0.53)	<0.001
Isokinetic knee extensors peak torque (60°/s)	69.0 (44.8–96.5)	55.3 (39.7–80.8)	87.8 (58.4–119.4)	<0.001
Relative knee extensors peak torque (60°/s/kg)	0.91 (0.59–1.26)	0.74 (0.52–1.05)	1.1 (0.78–1.44)	<0.001
Gait speed (m/s)	1.10 (0.94–1.24)	1.05 (0.89–1.2)	1.15 (0.99–1.28)	0.001
30-s arm curl (reps)	14 (12–17)	14 (12–17)	15 (12–17)	0.423
30-s chair stand (reps)	12 (9–14)	11 (9–14)	12 (10–14)	0.085

Notes: Data are expressed as medians (25th–75th percentile). Differences between males and females have been calculated by Mann–Whitney U test. Abbreviations: BMI, body mass index; reps, repetitions.

**Table 2:** Total participants’ characteristics by ACTN3 genotypes.

Variable (Units)	RR (N = 117)	RX (N = 152)	XX (N = 13)	*p*-Value	RR + RX (N = 269)	XX (N = 13)	*p*-Value	RR (N = 117)	RX + XX (N = 165)	*p*-Value
Gender (f/m, [%f])	60/57 [51.3% f]	81/71 [53.3% f]	8/5 [61.5% f]	0.771	141/128 [52.4% f]	8/5 [61.5% f]	0.520	60/57 [51.3% f]	89/76 [53.9% f]	0.660
Age (years)	68.1 (62.4–71.8)	67.1 (59.4–73.0)	72.3 (67.5–75.3)	0.134	67.4 (60.5–72.4)	72.3 (67.5–75.3)	0.045 *	68.1 (62.4–71.8)	67.4 (59.8–73.4)	0.809
Body weight (kg)	78.2 (70.4–8.3)	77.2 (70.7–86.0)	80.4 (72.5–83.8)	0.662	77.3 (70.6–86.7)	80.4 (72.5–83.8)	0.563	78.2 (70.4–8.3)	77.3 (71.0–85.5)	0.561
Height (m)	1.64 (1.58–1.71)	1.64 (1.58–1.71)	1.65 (1.62–1.75)	0.309	1.64 (1.58–1.71)	1.65 (1.62–1.75)	0.128	1.64 (1.58–1.71)	1.64 (1.58–1.72)	0.640
BMI (kg/m^2^)	29.3 (26.0–33.0)	28.8 (26.6–32.1)	27.5 (25.0–30.8)	0.598	29.0 (26.5–32.4)	27.45 (25.0–30.8)	0.406	29.3 (26.0–33.0)	28.8 (26.5–32.0)	0.468
Skeletal muscle mass (kg)	25.9 (22.7–30.4)	25.6 (22.1–30.2)	27.2 (24.7–32.4)	0.438	25.8 (22.5–30.3)	27.2 (24.7–32.4)	0.214	25.9 (22.7–30.4)	25.7 (22.6–30.6)	0.925
Fat mass (kg)	27.8 (20.8–36.2)	28.6 (22.3–35.2)	28.3 (19.5–34.1)	0.970	28.4 (21.6–35.9)	28.3 (19.5–34.1)	0.854	27.8 (20.8–36.2)	28.6 (22.4–35.0)	0.844
Body fat percentage (%)	36.4 (28.5–45.0)	37.4 (29.6–44.0)	38.7 (24.5–41.8)	0.957	36.8 (29.2–44.3)	38.7 (24.5–41.8)	0.766	36.4 (28.5–45.0)	37.5 (29.7–44.0)	0.964
Handgrip strength (kg)	29.0 (24.4–37.2)	29.7 (23.3–37.3)	32.0 (26.6–38.4)	0.673	29.2 (23.9–37.2)	32.0 (26.6–38.4)	0.396	29.0 (24.4–37.2)	30.0 (23.5–37.6)	0.673
Relative handgrip strength (kg/kg)	0.38 (0.31–0.48)	0.39 (0.31–0.47)	0.36 (0.32–0.50)	0.810	0.39 (0.31–0.47)	0.36 (0.32–0.50)	0.667	0.38 (0.31–0.48)	0.39 (0.31–0.47)	0.578
Isokinetic knee extensors peak torque (60°/s)	70.6 (44.5–94.0)	68.3 (43.8–97.1)	68.7 (49.7–98.8)	0.635	69.2 (44.1–96.3)	68.7 (49.7–98.8)	0.580	70.6 (44.5–94.0)	68.6 (45.0–97.4)	0.388
Relative isokinetic knee extensors peak torque (60°/s/kg)	0.89 (0.58–1.19)	0.94 (0.60–1.30)	0.88 (0.65–1.18)	0.537	0.91 (0.58–1.28)	0.88 (0.65–1.18)	0.817	0.89 (0.58–1.19)	0.92 (0.62–1.30)	0.265
Gait speed (m/s)	1.08 (0.94–1.23)	1.12 (0.94–1.25)	1.09 (1.00–1.33)	0.682	1.11 (0.94–1.24)	1.09 (1.00–1.33)	0.598	1.08 (0.94–1.23)	1.12 (0.94–1.25)	0.434
30-s biceps curl (reps)	14 (12–17)	15 (12–17)	14 (13–17)	0.933	15.00 (12–17)	14 (13–17)	0.961	14 (12–17)	15 (12–17)	0.711
30-s chair stand (reps)	14 (9–14)	12 (9–14)	12 (9–15)	0.958	12 (9–14)	12 (9–15)	0.982	14 (9–14)	12 (9–14)	0.770

Notes: Data are expressed as medians (25th–75th percentile), absolute numbers (percentages). Differences between genotypes were determined by Kruskal–Wallis or Mann–Whitney U test. Asterisks denote significant differences with * *p* < 0.05. Abbreviations: f, females; m, males; BMI, body mass index; reps, repetitions.

**Table 3:** Female participants’ characteristics by ACTN3 genotypes.

Variable (Units)	RR (N = 60)	RX (N = 81)	XX (N = 8)	*p*-Value	RR + RX (N = 141)	XX (N = 8)	*p*-Value	RR (N = 60)	RX + XX (N = 89)	*p*-Value
Age (years)	64.5 (58.1–69.0)	65.6 (56.1–70.2)	71.3 (61.1–73.3)	0.210	64.7 (56.8–69.8)	71.3 (61.1–73.3)	0.094	64.5 (58.1–69.0)	66.2 (56.8–71.1)	0.380
Body mass (kg)	77.1 (68.2–87.2)	76.0 (68.1–80.9)	82.1 (72.6–87.9)	0.203	76.4 (68.2–83.5)	82.1 (72.6–87.9)	0.135	77.1 (68.2–87.2)	76.8 (68.5–82.2)	0.507
Height (m)	1.59 (1.54–1.62)	1.59 (1.53–1.63)	1.64 (1.60–1.65)	0.079	1.59 (1.53–1.62)	1.64 (1.60–1.65)	0.025 *	1.59 (1.54–1.62)	1.59 (1.54–1.63)	0.794
BMI (kg/m^2^)	31.2 (26.8–34.8)	29.9 (27.5–33.8)	30.2 (28.0–32.9)	0.664	30.2 (27.4–34.2)	30.2 (28.0–32.9)	0.940	31.2 (26.8–34.8)	29.9 (27.5–33.6)	0.368
Skeletal muscle mass (kg)	23.4 (21.3–25.0)	22.9 (20.7–25.3)	24.9 (23.3–27.0)	0.153	23.1 (21.0–25.2)	24.9 (23.3–27.0)	0.060	23.4 (21.3–25.0)	23.3 (20.9–25.4)	0.920
Fat mass (kg)	35.0 (25.0–41.6)	31.6 (26.8–39.1)	33.2 (29.0–40.6)	0.514	32.4 (25.9–39.4)	33.2 (29.0–40.6)	0.649	35.0 (25.0–41.6)	31.7 (27.3–39.1)	0.342
Body fat percentage (%)	44.3 (37.3–48.7)	42.8 (37.9–47.8)	40.6 (39.1–46.6)	0.656	43.5 (37.8–48.3)	40.6 (39.1–46.6)	0.743	44.3 (37.3–48.7)	42.8 (38.0–47.6)	0.365
Handgrip strength (kg)	25.9 (22.5–28.6)	27.7 (21.1–28.5)	27.7 (22.7–31.4)	0.326	25.3 (21.7–28.6)	27.7 (22.7–31.4)	0.257	25.9 (22.5–28.6)	25.0 (21.3–28.8)	0.460
Relative handgrip strength (kg/kg)	0.34 (0.27–0.40)	0.32 (0.28–0.38)	0.34 (0.30–36)	0.871	0.33 (0.27–0.39)	0.34 (0.30–36)	0.880	0.34 (0.27–0.40)	0.32 (0.29–0.38)	0.642
Isokinetic knee extensors peak torque (60°/s)	51.5 (37.4–78.0)	55.3 (38.8–81.6)	76.5 (56.1–99.4)	0.075	54.8 (38.8–79.1)	76.5 (56.1–99.4)	0.024 *	51.5 (37.4–78.0)	55.6 (39.7–84.3)	0.493
Relative isokinetic knee extensors peak torque (60°/s/kg)	0.73 (0.51–1.03)	0.72 (0.49–1.07)	0.97 (0.79–1.19)	0.204	0.72 (0.50–1.04)	0.97 (0.79–1.19)	0.084	0.73 (0.51–1.03)	0.77 (0.52–1.10)	0.437
Gait speed (m/s)	1.04 (0.94–1.18)	1.06 (0.89–1.21)	1.23 (0.86–1.41)	0.537	1.05 (0.89–1.19)	1.23 (0.86–1.41)	0.272	1.04 (0.94–1.18)	1.06 (0.89–1.23)	0.687
30-s arm curl (reps)	15 (11–18)	14 (12–16)	15 (13–20)	0.597	14 (12–17)	15 (13–20)	0.368	15 (11–18)	14 (12–17)	0.777
30-s chair stand (reps)	11 (9–14)	11 (9–14)	13 (11–15)	0.406	11 (9–14)	13 (11–15)	0.210	11 (9–14)	12 (9–14)	0.817

Notes: Data are expressed as medians (25th–75th percentile). Differences between genotypes were determined by Kruskal–Wallis or Mann–Whitney U test. Asterisks denote significant differences with * *p* < 0.05. Abbreviations: f, females; m, males; BMI, body mass index; reps, repetitions.

**Table 4:** Male participants’ characteristics by ACTN3 genotypes.

Variable (Units)	RR (N = 57)	RX (N = 71)	XX (N = 5)	*p*-Value	RR + RX (N = 128)	XX (N = 5)	*p*-Value	RR (N = 57)	RX + XX (N = 76)	*p*-Value
Age (years)	70.1 (67.3–73.7)	69.9 (64.8–74.6)	73.3 (69.8–78.9)	0.305	70.1 (66.2–74.1)	73.3 (69.8–78.9)	0.141	70.1 (67.3–73.7)	70.4 (65.6–74.7)	0.845
Body mass (kg)	78.6 (73.3–88.5)	80.2 (73.8–88.1)	78.5 (72.5–81.7)	0.778	80.0 (73.3–88.4)	78.5 (72.5–81.7)	0.489	78.6 (73.3–88.5)	80.0 (73.8–87.9)	0.973
Height (m)	1.71 (1.65–1.75)	1.72 (1.67–1.75)	1.76 (1.73–1.80)	0.082	1.71 (1.66–1.75)	1.76 (1.73–1.80)	0.049 *	1.71 (1.65–1.75)	1.72 (1.67–1.75)	0.169
BMI (kg/m^2^)	28.6 (25.2–30.2)	27.4 (25.4–30.4)	24.8 (24.0–25.8)	0.077	28.1 (25.4–30.3)	24.8 (24.0–25.8)	0.026 *	28.6 (25.2–30.2)	27.3 (24.9–30.0)	0.428
Skeletal muscle mass (kg)	30.5 (27.0–34.4)	30.3 (26.8–34.6)	32.9 (31.4–35.9)	0.385	30.4 (27.0–34.5)	32.9 (31.4–35.9)	0.172	30.5 (27.0–34.4)	30.9 (27.4–34.8)	0.653
Fat mass (kg)	25.6 (19.5–29.0)	23.8 (17.9–29.9)	14.0 (12.9–24.9)	0.193	24.4 (18.9–29.4)	14.0 (12.9–24.9)	0.071	25.6 (19.5–29.0)	23.8 (16.8–29.6)	0.635
Body fat percentage (%)	30.2 (25.8–35.1)	30.4 (23.5–35.3)	18.97 (17.2–30.5)	0.179	30.4 (24.8–35.1)	19.0 (17.2–30.5)	0.067	30.2 (25.8–35.1)	30.1 (22.9–34.9)	0.540
Handgrip strength (kg)	36.6 (29.5–41.1)	36.9 (31.1–45.2)	38.2 (36.9–43.0)	0.208	36.8 (30.6–42.9)	38.2 (36.9–43.0)	0.381	36.6 (29.5–41.1)	37.5 (31.3–45.1)	0.095
Relative handgrip strength (kg/kg)	0.45 (0.34–0.52)	0.45 (0.40–0.54)	0.52 (0.46–0.57)	0.194	0.45 (0.39–0.53)	0.52 (0.46–0.57)	0.181	0.45 (0.34–0.52)	0.46 (0.41–0.54)	0.152
Isokinetic knee extensors peak torque (60°/s)	86.3 (54.9–116.6)	90.1 (62.4–123.4)	50.5 (46.8–115.1)	0.225	88.9 (62.1–119.6)	50.5 (46.8–115.1)	0.232	86.3 (54.9–116.6)	88.3 (62.1–122.6)	0.305
Relative isokinetic knee extensors peak torque (60°/s/kg)	1.06 (0.75–1.41)	1.12 (0.90–0.15)	0.66 (0.62–1.44)	0.225	1.10 (0.82–1.45)	0.66 (0.62–1.44)	0.282	1.06 (0.75–1.41)	1.11 (0.87–1.49)	0.252
Gait speed (m/s)	1.15 (0.93–1.28)	1.16 (0.97–1.29)	1.08 (1.06–1.14)	0.585	1.16 (0.97–1.29)	1.08 (1.06–1.14)	0.445	1.15 (0.93–1.28)	1.15 (1.04–1.29)	0.576
30-s arm curl (reps)	14 (12–16)	15 (12–18)	13 (12–15)	0.308	15 (12–17)	13 (12–15)	0.293	14 (12–16)	15 (12–18)	0.356
30-s chair stand (reps)	12 (10–14)	12 (10–15)	9 (6–13)	0.167	12 (10–14)	9 (6–13)	0.117	12 (10–14)	12 (10–15)	0.438

Notes: Data are expressed as medians (25th–75th percentile). Differences between genotypes were determined by Kruskal–Wallis or Mann–Whitney U test. Asterisks denote significant differences with * *p* < 0.05. Abbreviations: f, females; m, males; BMI, body mass index; reps, repetitions.

## Data Availability

The data presented in this study are available on request from the corresponding author.
